# Temporal trends and patterns in atrial fibrillation incidence: A population-based study of 3·4 million individuals

**DOI:** 10.1016/j.lanepe.2022.100386

**Published:** 2022-04-25

**Authors:** Jianhua Wu, Ramesh Nadarajah, Yoko M. Nakao, Kazuhiro Nakao, Chris Wilkinson, Mamas A. Mamas, A. John Camm, Chris P. Gale

**Affiliations:** a Leeds Institute for Cardiovascular and Metabolic Medicine, University of Leeds, 6 Clarendon Way, Leeds LS2 9DA, UK; b School of Dentistry, University of Leeds, Leeds, UK; c Leeds Institute for Data Analytics, University of Leeds, UK; d Department of Cardiology, Leeds Teaching Hospitals NHS Trust, Leeds, UK; e Faculty of Medical Sciences, Population Health Sciences Institute, Newcastle University, UK; f Keele Cardiovascular Research Group, Institute for Prognosis Research, University of Keele, Keele, UK; g Molecular and Clinical Sciences Research Institute, St George's University of London, UK

**Keywords:** Atrial fibrillation, Electronic health records, Incidence, Multimorbidity

## Abstract

**Background:**

Population-based studies of atrial fibrillation (AF) incidence are needed to inform health-service planning, but evidence is conflicting. We assessed trends of AF incidence in a large general population cohort from England.

**Methods:**

We used linked primary and secondary electronic health records of 3.4 million individuals. Eligible patients aged 16 years and older contributed data between Jan 2 1998 and Dec 31 2017. For patients with incident AF, we extracted baseline characteristics, comorbidities, socioeconomic status and geographic region. We calculated standardised rates by applying direct age and sex standardisation to the 2013 European Standard Population. We applied year-specific, age-specific and sex-specific incidence to UK census mid-year population estimates for yearly total incident AF.

**Findings:**

Comparing 2017 to 1998 standardised AF incidence increased by 30% (322 vs. 247 per 100 000 person-years; adjusted incidence ratio [IRR] 1·30, 95% CI 1·27–1·33). Absolute number of incident AF increased by 72% (202 333 vs. 117 880), due to an increasing number of older persons. Comorbidity burden at diagnosis of AF increased (3·74 [SD 2·29] vs 2·58 [1·83]; adjusted difference 1·26, 95% CI 1·14–1·39). The age of AF diagnosis declined in the most deprived individuals compared to the most affluent (adjusted difference 0·74 years, 0·62–0·88). Across the study period, age-standardised incidence was higher in men than women (IRR 1·49; 95% CI 1·46–1·52), and men were younger at diagnosis (adjusted difference 5·53 years; 95% CI 5·36 to 5·69). Socioeconomically deprived individuals had more comorbidities and a higher incidence of AF than the most affluent individuals (IRR 1·20; 95% CI 1·15–1·24).

**Interpretation:**

In England AF incidence has increased, and the socioeconomic gradient in age at diagnosis and comorbidity burden widened. This changing burden requires policy-based interventions to achieve health equity.

**Funding:**

British Heart Foundation and National Institute for Health Research.

**Research in context**

**Evidence before this study**

We searched Medline and Embase for reports published in English from inception to December 2021 that included “atrial fibrillation” and “incidence” in their title. We also reviewed clinical practice guidelines and reference lists of selected reports.

Estimates of atrial fibrillation (AF) incidence rates and trends over time are inconsistent due to methodological inconsistency between studies. Studies frequently referred to populations restricted by age or specific morbidities, or limited case finding to either primary or secondary care. Furthermore, reports differed in their approach to standardisation, such that some presented crude rates and others standardised to census populations. Very few referred to a standard population, estimates of incidence in high income countries vary by over 12-fold, and comparison between studies is challenging. We found no study that provided a comprehensive assessment of AF incidence by comorbidity burden, socioeconomic status and geographic region.

**Added value of this study**

Our study provides contemporary insights into the burden of incident AF in England and its variation over time by age, sex, socioeconomic status and geographic region. We report standardised incidence rates derived from a large, representative general population that can act as the standard for international comparison. We demonstrate that between 1998 and 2017 AF incidence has increased across all age groups and both sexes in England, and has now reached the same magnitude as the most recent estimate for heart failure. In fact, with population growth and ageing the burden of AF outstrips the combined incidence of breast, prostate, lung and bowel cancer in 2021. We also provide evidence that over time there has been a substantial rise in the number of associated comorbidities at first presentation of AF, and that a disparity has developed in age at first presentation across socioeconomic strata.

**Implications of all evidence available**

Whilst improvements have been made in heart failure and ischaemic heart disease prevention, the incidence of AF has risen to reach a similar level to heart failure and outstrip the combined four most common causes of cancer in England. Countries across Europe, North America and Australasia have similar population structures and temporal changes and are likely to have a similar burden. Notably, the increasing number of older, more comorbid individuals with AF could lead to an increased burden of stroke and hospitalisations. The observed disparities in AF incidence and age of diagnosis by sex and socioeconomic status highlight opportunities for more targeted prevention strategies and resource utilisation to aim for health equity and curtail the rising tide of cases and associated adverse sequelae.

Alt-text: Unlabelled box

## Introduction

Atrial fibrillation (AF) is the most common sustained cardiac arrhythmia worldwide; confers an increased risk of stroke, heart failure, cognitive decline and death, and is associated with quantifiable impairment in quality of life.^1^ Once diagnosed most patients will require lifelong-treatment; rate or rhythm control for symptom relief and oral anticoagulation to mitigate the elevated risk of stroke.^1^ Policies to direct public health initiatives and health service delivery for AF require robust, contemporary population-level disease incidence estimates. Information is required about standardised rates, which describe disease incidence independent of changes in population, and absolute numbers of people affected, which will tell the direct impact on health services.

Reports of temporal trends in AF incidence in Western countries are inconsistent due to methodological inconsistency (supplementary Table S1). Most studies demonstrate an increase in incidence,^2^, ^3^, ^4^ whilst others suggest that it remains steady,^5^^,^^6^ and one even reports that it has started to decline in recent years.^7^ Age distributions of study populations have varied from 18 years and older to 65 years and older,^2^^,^^5^ and some studies excluded individuals with known valvular heart disease.^8^^,^^9^ Amongst population studies some rely on health claims data, which are not directly comparable with estimates from medical record review.^5^^,^^10^ Moreover, case identification has often been restricted to either those made during hospital attendances,^6^^,^^7^ or in primary care.^11^^,^^12^ Only by considering presentations across the spectrum of acute and chronic care for all adults can the burden of AF be captured and resources directed to improve care and outcomes.

Furthermore, reports of AF incidence have differed in their approach to standardisation: some present crude rates,^13^^,^^14^ others adjust for differences in the denominator population,^3^^,^^12^ with the majority standardising to census population.^2^^,^^4^, ^5^, ^6^^,^^8^^,^^10^ However, the scarcity of reports referring to a standard population renders comparisons between studies challenging.^7^ As for the absolute number of cases of incident AF country-specific estimates are rarely reported - last so in the United Kingdom (UK) in 2015.^8^ Additionally, long-term temporal trends on heterogeneity in incident AF according to socioeconomic deprivation,^12^ comorbidity burden,^3^^,^^4^ and geographic region have yet to be investigated in a comprehensive nationwide study.

To address this knowledge gap we used a large longitudinal database of linked primary and secondary care records from a representative sample of the English population to assess trends in the crude and standardised atrial fibrillation incidence by sex, age, socioeconomic status and region. We also investigated the comorbidity profile of patients over almost two decades.

## Methods

### Data source

We used electronic health records from the Clinical Practice Research Datalink (CPRD) from Jan 1, 1985, to Nov 30, 2018. The CPRD database contains anonymised patient data from approximately 7% of the UK population and is broadly representative in terms of age, sex, and ethnicity.^15^ CPRD is one of the largest databases of longitudinal medical records from primary care in the world. Diagnostic coding for AF in CPRD has been shown to be consistent and valid, with a positive predictive value of 98%.^16^ The dataset used for this analysis was primary care records from CPRD that had been linked to secondary care admission records from Hospital Episodes Statistics Admitted Patient Care (HES-APC) data. Linkage is available for a subset of English practices from Jan 2, 1998, covering approximately 50% of all CPRD records. Previous research has demonstrated the representativeness of patients eligible for linkage in terms of age, gender and geography.^17^ Scientific approval for this study was given by the CPRD Independent Scientific Advisory Committee (ISAC).

### Study population

Participants were men and women aged 16 years and older, contributing to data between Jan 2, 1998, and Dec 31, 2017. Patients were eligible for inclusion if their record was labelled as acceptable by CPRD quality control,^15^ approved for CPRD and Hospital Episodes Statistics linkage, and if they were registered with their general practice for at least 12 months.

For incidence calculations, we excluded all individuals who had a diagnosis of AF before the study start date (Jan 1, 1985, to Jan 1, 1998, in primary and secondary care records), or within the first 12 months of registration with their general practice.

### Case identification and categorisation

To identify AF diagnoses, we used a list of 24 diagnostic codes from hospital (International Classification of Diseases, tenth revision [ICD-10]) and primary care (Read) coding schemes which included both AF and atrial flutter (supplementary Table S2). We defined incident AF diagnosis as the first record of AF in primary care or hospital admission records from any diagnostic position. According to the source of the first recording, cases were allocated to either primary care (CPRD) or hospital-based (HES-APC, subdivided as primary or non-primary discharge diagnosis).^8^

### Patient characteristics

For patients with incident AF, we extracted the most recent measurement of baseline characteristics within 1 year of a diagnosis of AF i.e., systolic and diastolic blood pressure, smoking status, and body mass index (BMI) - from electronic health records.

We also extracted information about comorbidities, socioeconomic status, ethnicity, and geographic region. To describe comorbidities, we selected 18 common chronic conditions: anaemia, cancer, chronic kidney disease, chronic obstructive pulmonary disease, dementia, depression, diabetes, dyslipidaemia, heart failure, hypertension, ischaemic heart disease, obesity, obstructive sleep apnoea, osteoarthritis, peripheral arterial disease, stroke or transient ischaemic attack, thyroid disease and valvular heart disease. For each condition, we report prevalence as the percentage of patients with a diagnosis recorded in their primary care or hospital discharge record, before their first diagnosis of AF. Diagnosis code lists from the CALIBER code repository were used for each condition. We used the Index of Multiple Deprivation (IMD) 2015 quintile to describe socioeconomic status.

### Statistical analyses

Baseline characteristics are presented as frequencies (%) for categorical data, medians and IQR for non-normally distributed continuous data, or means and SD for normally distributed continuous data. Data are stratified by sex, socioeconomic quintile, and year of diagnosis. Frequencies and percentages for socioeconomic status, smoking, systolic and diastolic blood pressure, heart rate and body mass index were reported using complete cases.

We computed age-specific, sex-specific, and year- specific incidence by dividing the number of incident cases by the number of patient-years in the cohort. Time at risk was restricted to number of days alive, in people aged 16 years and older, who were registered with a general practice for over 12 months during the practice's up-to-standard periods.

To calculate standardised rates, we applied direct age and sex standardisation to the 2013 European Standard Population using 5-year age bands up to 90 years of age. The European Standard Population is an artificial population structure, designed and published by the statistical office of the European Union (Eurostat), to allow the calculation of age-standardised and sex-standardised rates that are comparable across regions and time.^18^

The estimated absolute number of yearly new diagnoses of AF were inferred by applying year-specific, age-specific, and sex-specific incidence to UK census mid-year population estimates, using 5-year age bands up to 90 years of age.

We used Poisson regression models to examine overall and category-specific incidence ratios and corresponding 95% confidence intervals (CI). When applicable, we adjusted models for time, age, sex, region, and socioeconomic status.

Study findings are reported in accordance with the Reporting of studies Conducted using Observational Routinely-collected health Data (RECORD) recommendations.^19^ We used R (version 4.0) to perform statistical analysis.

### Role of funding source

The funders of the study had no role in study design, data collection, data analysis, data interpretation, or writing the report.

## Results

A total of 3,383,148 patients aged 16 years or over contributed data between Jan 2, 1998, and Dec 31, 2017. We excluded all individuals diagnosed with AF within the first 12 months of registration with their general practice (5806 patients) for incidence calculations, leading to 3,377,342 eligible patients and a total of 32,044,121 patient-years at risk. Of those, 84,870 developed AF during the study period.

Patient characteristics stratified by sex, socioeconomic status and time period categories are shown in the Table 1. The mean age at AF diagnosis was 78·0 years (SD 12·3) and 48·6% of those with incident AF were women. From 1998 to 2017, the mean age at diagnosis decreased slightly from 76·1 years (SD 11·6) to 75·8 years (SD 12·6; adjusted difference -0·26 years, 95% CI -0·53–0·00; Table 1).

**Table 1:** Characteristics of patients with incident atrial fibrillation.

	All patients		Sex			Socioeconomic status			Time period	
			Women	Men		SES 1	SES 5		1998–2002	2013–2017
	*n* = 84870		*n* = 41269	*n* = 43601		*n* = 18065	*n* = 13091		*n* = 14395	*n* = 17657
Age (years)	75.99 (12·31)		78·83 (11·49)	73·30 (12·45)		75·76 (12·38)	75·25 (12·62)		76·07 (11·59)	75·80 (12·62)
Men	43601 (51·4)		0 (0·0)	43601 (100·0)		9687 (53·6)	6516 (49·8)		7184 (49·9)	9326 (52·8)
Ethnicity (White)	77835 (93·4)		37689 (92·8)	40146 (94·0)		16569 (93·9)	11962 (92·7)		12062 (86·6)	16502 (95·4)
Socioeconomic status quintile										
1 (least deprived)	18065 (21·3)		8378 (20·3)	9687 (22·2)		18065 (100·0)	··		2678 (18·6)	4146 (23·5)
2	18293 (21·6)		8739 (21·2)	9554 (21·9)		··	··		3103 (21·6)	3712 (21·0)
3	19333 (22·8)		9414 (22·8)	9919 (22·8)		··	··		3435 (23·9)	3902 (22·1)
4	16052 (18·9)		8143 (19·7)	7909 (18·1)		··	··		2675 (18·6)	3364 (19·1)
5 (most deprived)	13091 (15·4)		6575 (15·9)	6516 (15·0)		··	13091 (100·0)		2493 (17·3)	2531 (14·3)
Ever smoker	43175 (55·4)		16487 (43·9)	26688 (66·2)		8393 (50·1)	7526 (62·6)		3452 (33·7)	11023 (62·9)
Systolic blood pressure (mm Hg)	137·01 (16·60)		138·90 (17·02)	135·24 (16·01)		136·85 (16·07)	136·74 (17·03)		144·65 (19·43)	133·24 (14·34)
Diastolic blood pressure (mm Hg)	77·36 (9·12)		77·51 (8·98)	77·22 (9·25)		77·56 (8·80)	77·14 (9·36)		80·92 (9·64)	75·61 (8·50)
Heart rate (bpm)	78·07 (16·42)		79·72 (16·34)	76·59 (16·35)		77·12 (16·59)	79·39 (16·46)		80·75 (17·91)	76·78 (15·29)
Body mass index (kg/m2)	27·73 (6·29)		27·50 (6·94)	27·91 (5·70)		27·29 (5·80)	28·26 (6·91)		26·84 (5·74)	28·23 (6·60)
Anaemia	11965 (14·1)		7168 (17·4)	4797 (11·0)		2232 (12·4)	2191 (16·7)		1536 (10·7)	2958 (16·8)
Cancer	18321 (21·6)		8475 (20·5)	9846 (22·6)		4215 (23·3)	2549 (19·5)		1965 (13·7)	4901 (27·8)
Chronic kidney disease	15365 (18·1)		8354 (20·2)	7011 (16·1)		3133 (17·3)	2523 (19·3)		425 (3·0)	4848 (27·5)
Chronic obstructive pulmonary disease	20951 (24·7)		10183 (24·7)	10768 (24·7)		3684 (20·4)	4175 (31·9)		3442 (23·9)	4499 (25·5)
Dementia	3202 (3·8)		1961 (4·8)	1241 (2·8)		404 (2·2)	335 (2·6)		270 (1·9)	1089 (6·2)
Depression	13380 (15·8)		8210 (19·9)	5170 (11·9)		2476 (13·7)	2508 (19·2)		1970 (13·7)	3136 (17·8)
Diabetes	13982 (16·5)		6127 (14·8)	7855 (18·0)		2602 (14·4)	2588 (19·8)		1597 (11·1)	3855 (21·8)
Dyslipidaemia	12889 (15·2)		6119 (14·8)	6770 (15·5)		2717 (15·0)	2191 (16·7)		901 (6·3)	3799 (21·5)
Heart failure	17195 (20·3)		8505 (20·6)	8690 (19·9)		3063 (17·0)	3135 (23·9)		4285 (29·8)	2577 (14·6)
Hypertension	47350 (55·8)		24905 (60·3)	22445 (51·5)		9931 (55·0)	7417 (56·7)		6126 (42·6)	11094 (62·8)
Ischaemic heart disease	21789 (25·7)		8833 (21·4)	12956 (29·7)		4244 (23·5)	3805 (29·1)		4169 (29·0)	4072 (23·1)
Obesity	12104 (14·3)		5448 (13·2)	6656 (15·3)		2190 (12·1)	2288 (17·5)		890 (6·2)	3273 (18·5)
Obstructive sleep apnoea	1866 (2·2)		422 (1·0)	1444 (3·3)		404 (2·2)	335 (2·6)		129 (0·9)	610 (3·5)
Osteoarthritis	31154 (36·7)		17519 (42·5)	13635 (31·3)		6355 (35·2)	5118 (39·1)		4431 (30·8)	7129 (40·4)
Peripheral arterial disease	4131 (4·9)		1697 (4·1)	2434 (5·6)		654 (3·6)	832 (6·4)		452 (3·1)	1017 (5·8)
Thyroid disease	8213 (9·7)		6299 (15·3)	1914 (4·4)		1723 (9·5)	1278 (9·8)		966 (6·7)	2012 (11·4)
Stroke/transient ischaemic attack	12276 (14·5)		6062 (14·7)	6214 (14·3)		2464 (13·6)	2025 (15·5)		2274 (15·8)	2526 (14·3)
Valvular heart disease	5147 (6·1)		2402 (5·8)	2745 (6·3)		1119 (6·2)	783 (6·0)		454 (3·2)	1593 (9·0)
Three or more comorbidities	49372 (58·2)		25464 (61·7)	23908 (54·8)		9791 (54·2)	8334 (63·7)		6553 (45·6)	11730 (66·4)

Data are mean (SD) or n (%). Socioeconomic status (SES) refers to Index of Multiple Deprivation 2015 quintile, with SES 1 referring to the most affluent and SES 5 to the most deprived socioeconomic quintile. Number of comorbidities refers to any of the 18 conditions investigated. The missing percentages for ethnicity, socioeconomic status, smoking, systolic and diastolic blood pressure, heart rate and body mass index are 1·8%, 0·04%, 8·2%, 17·0%, 17·0%, 70·3%, and 53·4%, respectively. Frequencies and percentages for variables with missing values refers to complete cases.

**Figure 1:**
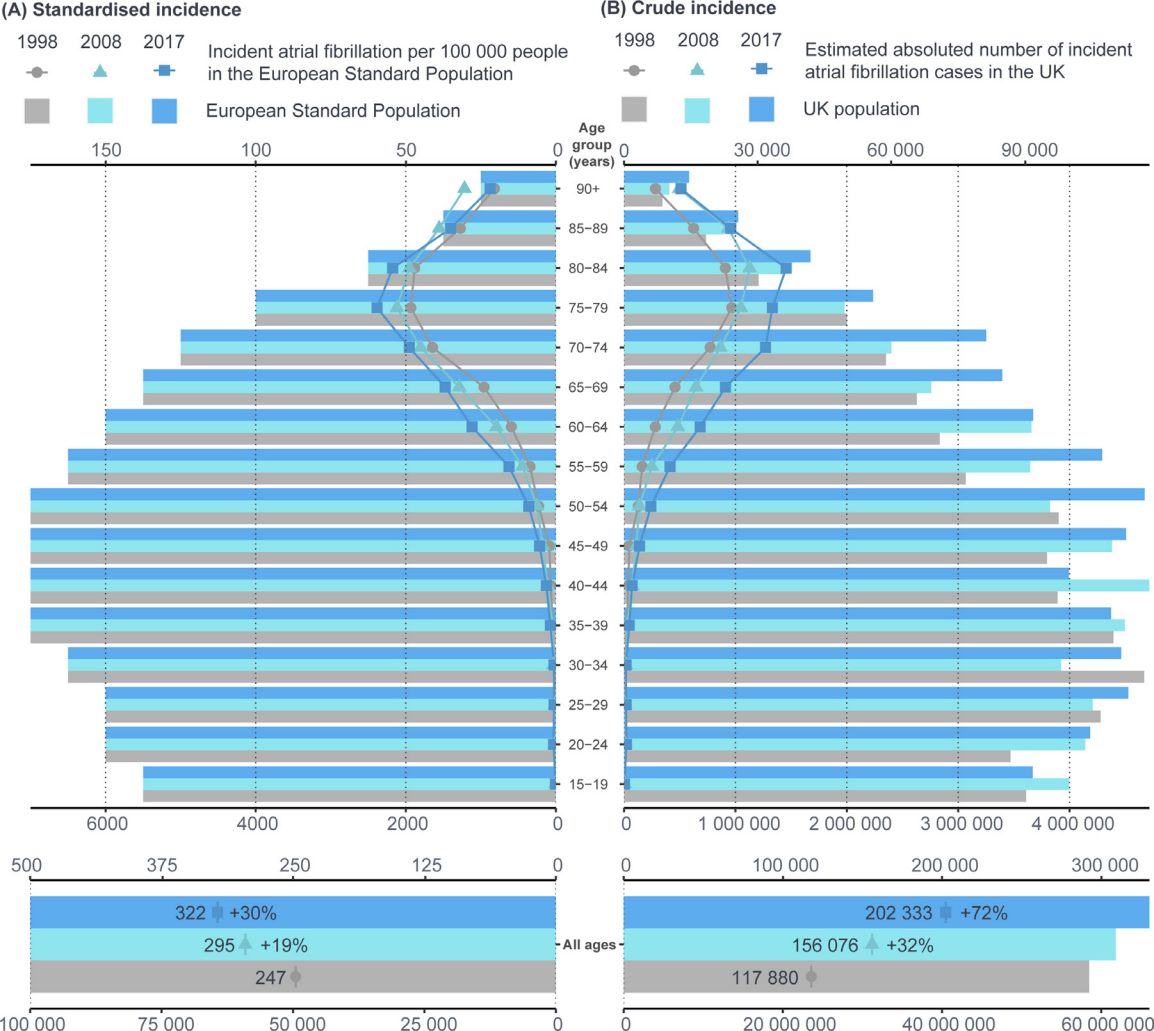
Overall and age-stratified atrial fibrillation incidence in 1998, 2008 and 2017. (A) Number of cases of incident atrial fibrillation per 100 000 people in the European Standard Population. (B) Estimated absolute number of cases of incident atrial fibrillation in the UK population (based on census mid-year estimates).

**Figure 2:**
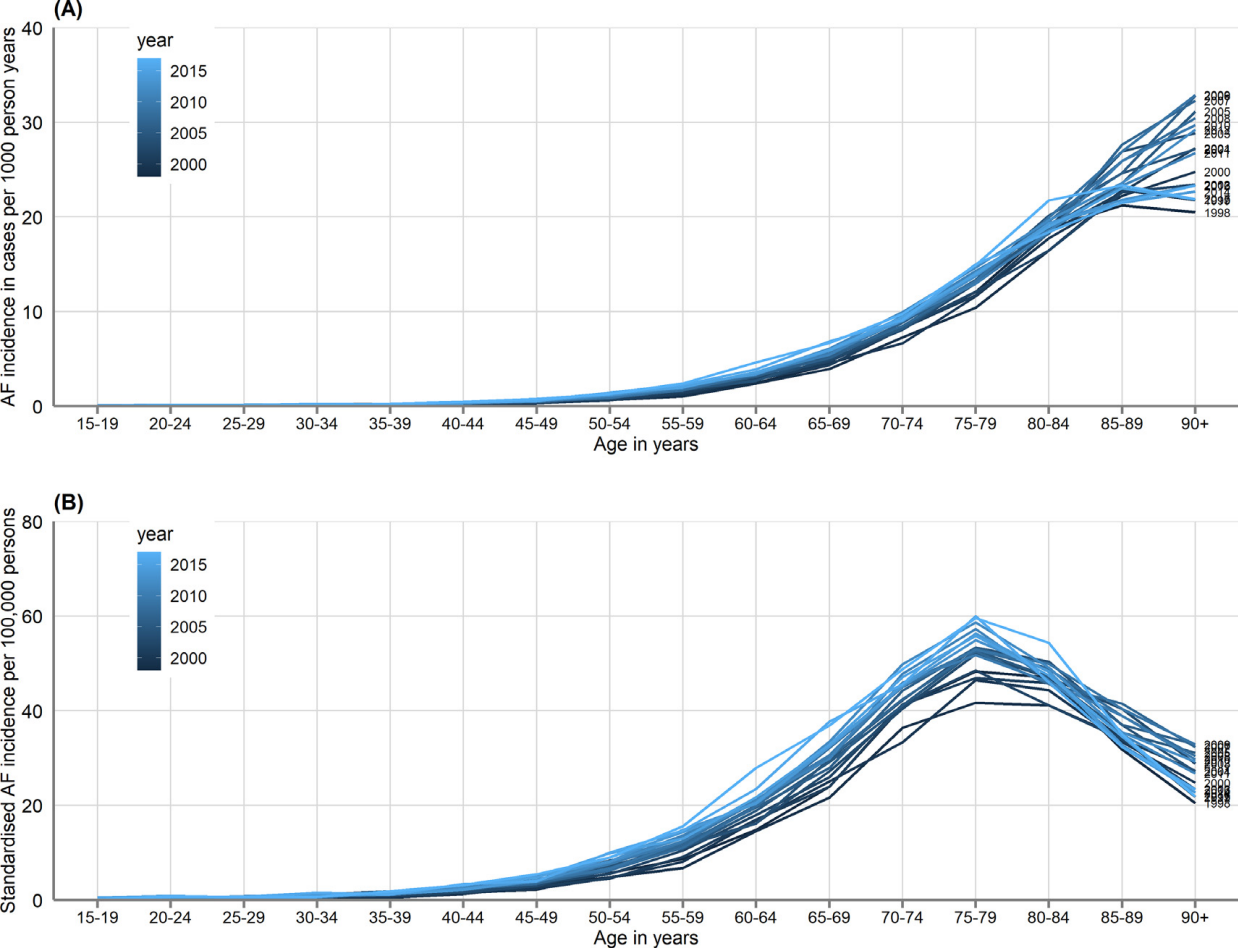
Atrial fibrillation incidence, by age-group and year of diagnosis. (A) Atrial fibrillation incidence per 1000 person years. (B) Standardised atrial fibrillation incidence per 100,000 persons using European Standard Population.

In models standardised for age and sex, AF incidence had increased by 30% in 2017 compared to 1998 (322 per 100,000 person-years vs. 247 per 100,000 person-years; adjusted incidence rate ratio [IRR] 1·30, 95% CI 1·27–1·33; Figure 1A). On time-trend analysis standardised incidence increased between 1998 and 2012 and plateaued thereafter (supplementary Figure S1). This overall increase was consistent across all age groups. Crude incidence increased with age whereas incidence standardised to the European Standard Population peaked in the 75–79 age group and thereafter declined as the number of people aged 80 years or older is much smaller in this artificial population than the UK population (Figures. 1, 2). Crude incidence increased by 47% from 250 per 100,000 people in 1998 to 367 per 100,000 people in 2017, with the increase steady over time due to population growth especially in older age groups (Figure 1B, supplementary Figure S1). The estimated absolute number of yearly new diagnoses of AF had increased by 72% in 2017 compared to 1998 (202,333 vs. 117,880).

Over time, the proportion of AF diagnosis that was first documented in hospital increased (1998, 51·9% to 2017, 58·1%) with a plateau from 2010 onwards; but the proportion of individuals with a primary AF discharge diagnosis decreased in a linear fashion (21·1% to 12·9%; supplementary Figure S2). Across the study period there was a U-shaped distribution in hospital-based AF diagnosis by age, with the youngest and oldest individuals most likely to be diagnosed first in hospital across both sexes (supplementary Figure S3A). Amongst hospital-based AF diagnoses, a lower proportion constituted the primary discharge diagnosis in older individuals compared to younger individuals (supplementary Figure S3B).

**Figure 3:**
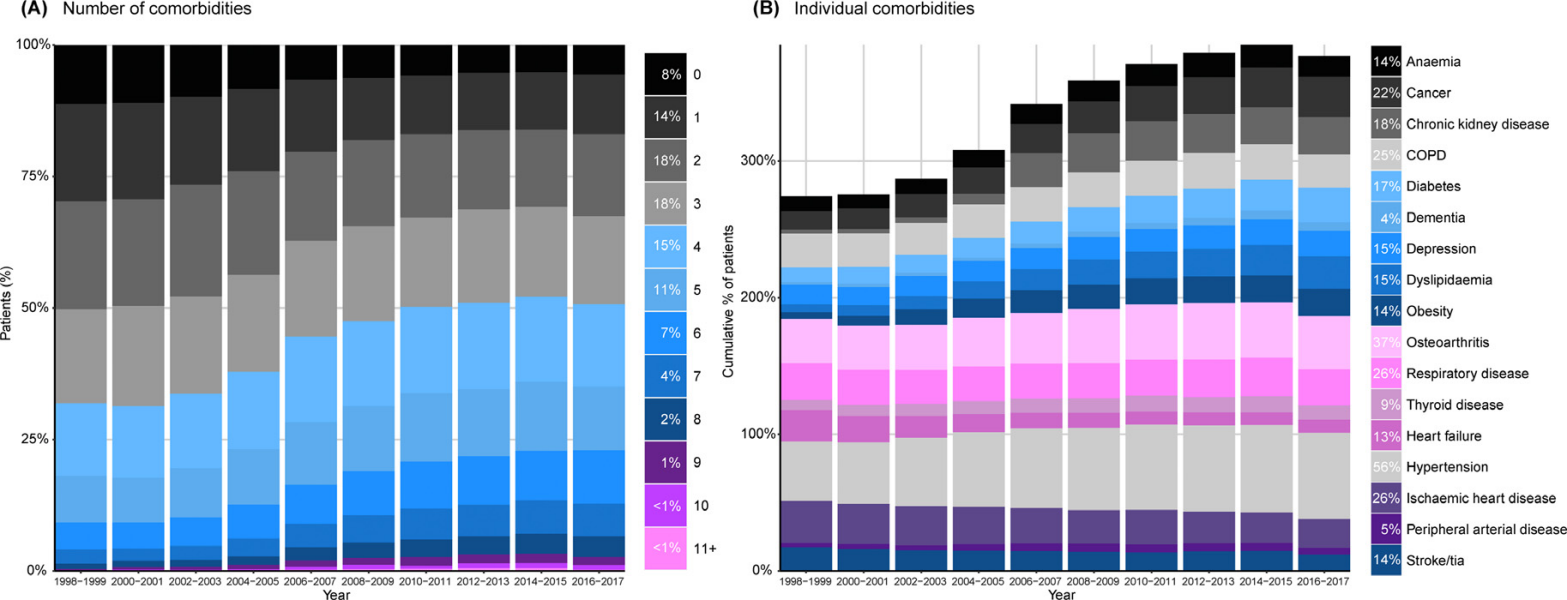
Temporal trends in comorbidities among patients diagnosed with incident atrial fibrillation, from 1998–2017. (A) Number of comorbidities, out of 18 major conditions, affecting patients with incident atrial fibrillation, over time. (B) Cumulative percentage of patients affected by individual comorbidities over time. COPD = chronic obstructive pulmonary disease; TIA = transient ischaemic attack.

The number of comorbidities at or before diagnosis of AF was high (mean 3·30 [SD 2·17]) and increased over time, from 2·58 (SD 1·83) in 1998 to 3·74 (SD 2·29) in 2017 (difference adjusted for age, sex and socioeconomic status 1·26, 95% CI 1·14–1·39; Figure 3a). Overall 60% of patients had three or more comorbidities, increasing from 48% in 1998 to 68% in 2017 (Table 1). Each of hypertension, osteoarthritis and ischaemic heart disease were prevalent in over a quarter of patients at or before diagnosis of AF. Over time, of comorbidities known to be associated with the development of AF, the prevalence of chronic kidney disease, valvular heart disease, diabetes, thyroid disease, obesity and hypertension increased whilst the prevalence of ischaemic heart disease and heart failure declined (Figure 3b, Table 1).

**Figure 4:**
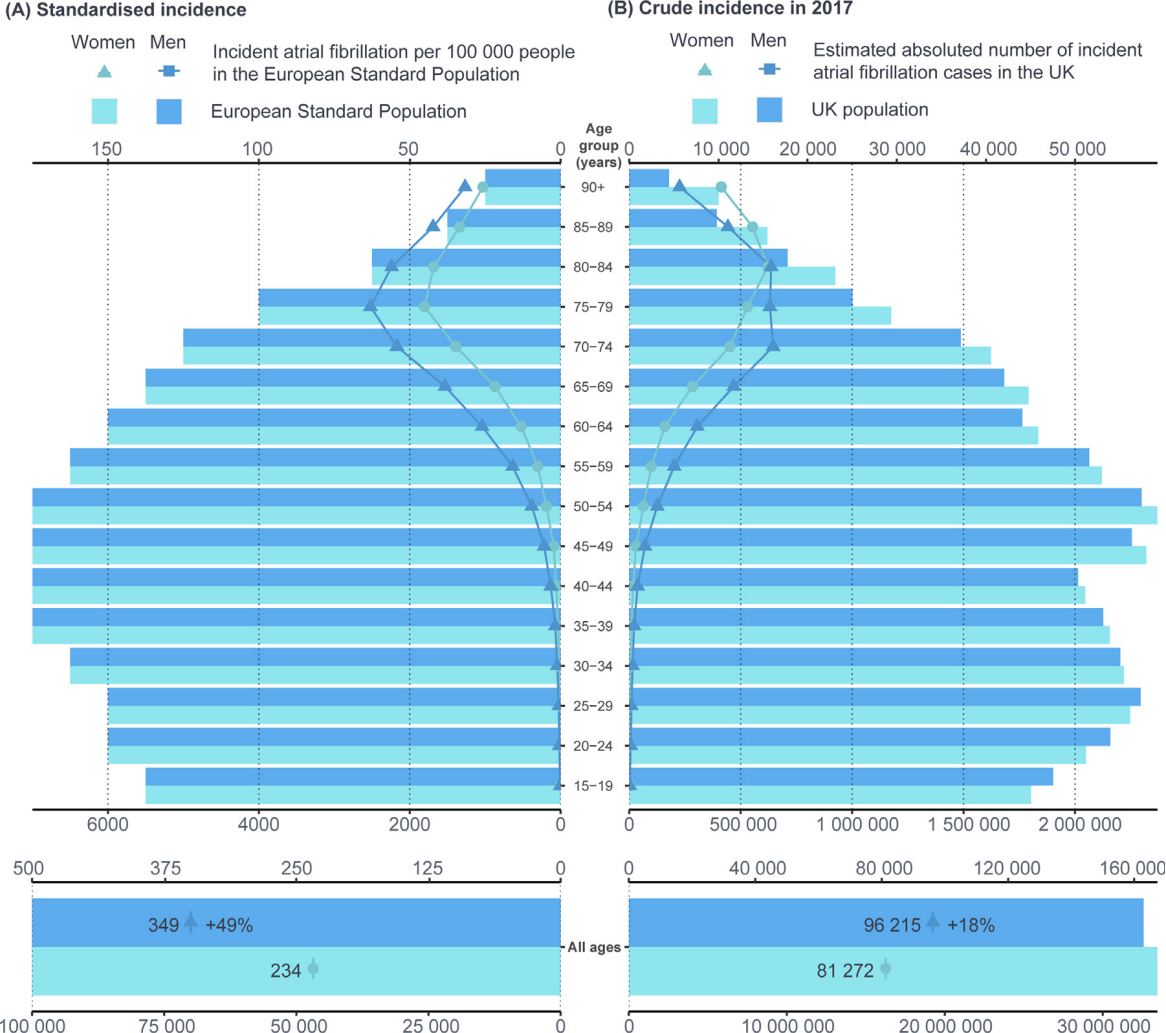
Overall and age-stratified atrial fibrillation incidence for women and men. Standardised atrial fibrillation (AF) incidence (A) presents cases in 100,000 persons from the European standard population. Crude incidence (B) presents estimated absolute number of cases in the United Kingdom (UK) population (2017 census mid-year estimates). Incidence rates were calculated overall years from 1998 to 2017.

The age standardised incidence of AF was higher in men than in women (IRR 1·49, 95% CI 1·46–1·52), which was consistent across age groups and remained stable over time (Figure 4, supplementary Figure S4). Because there was a higher number of women in older age groups the estimated total number of incident cases in 2017 was only 18% higher in men. Men were younger than women at date of diagnosis (mean age 73·3 years [SD 12·5] *vs* 78·8 years [SD 11·5]; adjusted difference 5·53, 95% CI 5·36–5·69; Table 1). Concurrently, men had a higher prevalence of smoking, diabetes, obstructive sleep apnoea and ischaemic heart disease than did women (Table 1).

At the same age and sex, patients in the most deprived socioeconomic quintile were more likely to experience AF (IRR 1·20, 95% CI 1·15–1·24) than were patients in the most affluent socioeconomic quintile; and this finding was consistent across all age groups and both sexes. Individuals who were most deprived were also more likely to have three or more comorbidities at baseline than the most affluent individuals (Table 1). In stratified time-trend analysis the increase in incidence, standardised by age and sex, did not differ between socioeconomic quintiles (supplementary Figure S5). However from 2008 onwards a difference developed in age at which AF was diagnosed by socioeconomic status (supplementary Figure S6), which was consistent across both men and women (supplementary Figure S7). Over time, the mean age at AF diagnosis decreased by 1·09 years (95% CI 0·41–1·77) amongst the most deprived but did not alter amongst the most affluent (0·35 years, 95% CI -0·20–0·90; adjusted difference 0·74, 95% CI 0·62–0·88).

The incidence standardised by age and sex increased in most regions over time with variation in the extent of increase. The greatest increases over time were seen in London (67% from 178 to 298 per 100,000 people), the West Midlands (50% from 238 to 356 per 100,000 people), and the South East (43% from 258 to 368 per 100,000 people); whereas the smallest increase was seen in the South West (9% from 248 to 270 per 100,000 people; supplementary Figure S8).

## Discussion

This large-scale, representative, population-based study provides several insights into the burden of incident AF in England. We report the first national estimate of AF incidence that refers to a standard population, thereby rendering a standard for international comparison, and provide a comprehensive assessment of variation in AF incidence over time, and by age, sex, socioeconomic status and geographic region.

The incidence rate of AF, standardised by age and sex, increased from 1998 to 2017, which contrasts with the pattern seen for myocardial infarction and heart failure, for both of which incidence decreased over a similar time period.^20^^,^^21^ It is possible that the decline in the incidence of these other two major cardiovascular conditions is related. National programmes of vascular checks to address key risk factors for ischaemic heart disease have been systematically embedded,^22^ and improvements in medical treatment of myocardial infarction has led to a lower burden of post-myocardial infarction ventricular dysfunction.^23^ Notably, our analysis estimates AF incidence standardised by age and sex as almost identical to the most recent estimate for heart failure (322 *vs* 332 per 100,000 people) derived from the same nationwide database.^20^ Thus, the incidence of AF may surpass the incidence of heart failure if current trends continue. In our stratified analysis the increase in incidence of AF was largely consistent across both sexes, all ages and socio-economic groups. By region there was a variation in the increase in incidence which may relate to differences in age, sex and deprivation between different regions, and change in the constituent population over time. It is notable that we report an increase in incidence rates standardised by age and sex, suggesting that factors other than an ageing population are contributing.

The total number of comorbidities at the time of AF diagnosis was high and increased over time. Specifically, several comorbidities which are known to be associated with the development of AF increased in prevalence, and the proportion of adults who had ever smoked almost doubled over time.^1^ Risk factor burden and having multiple comorbidities have a crucial role in lifetime risk of AF.^24^ With an optimal risk factor profile - that is, being unaffected by myocardial infarction, heart failure and following guideline recommendations on smoking, alcohol, body mass index, blood pressure, glycaemic control – the lifetime risk of AF for a person aged 55 years or older is reduced to about one in five, rather than one in three in the presence of at least one of those risk factors.^24^

In our study men and individuals who were most deprived had less favourable risk factor profiles than women and those who were most affluent. Sex differences in the risk of AF and the burden of risk factors between men and women corresponds with previous European data.^24^ Our time-trend analyses further show that there has been no substantial convergence of the incidence rates by socioeconomic status, and in fact the age gap between the most and least deprived groups at diagnosis of AF widened. The most deprived individuals in our study also had a higher incidence of ischaemic heart disease and heart failure than the most affluent, which are amongst the most frequent reasons for admission following an AF diagnosis.^25^ Moreover, it has been reported that individuals in the most deprived socioeconomic quintile have higher AF fatality compared to the most affluent individuals.^25^

Changes in clinical practice may have contributed to the observed increase in AF incidence over time through detection of AF cases that were historically not diagnosed. We found that the proportion of AF diagnosis made first in hospital increased over time but predominantly was not the primary reason for admission to hospital. Clinical thresholds for investigation for AF and documentation of diagnosis may have been altered by the torrent of evidence since the early 1990s for effectiveness of oral anticoagulation in stroke prophylaxis.^26^ Over the last two decades increased use of ECG recording has been observed in populations at risk of AF and correlates with increasing AF incidence.^4^

Standardised trends are necessary to compare changes in disease burden over time and place, but to service payers, providers, and researchers, it is also important to know the absolute numbers of patients who require treatment. In the context of an increase in standardised incidence there is an extraordinary 72% increase in the total number of new cases of AF. By comparison, the total number of new AF cases (202,333) outstrips the combined total number of cases of breast, prostate, lung and bowel cancer in 2021 (199,608).^27^ The increase predominantly results from the increasing number of older people within the population, which portends a significant rise in total cases across Western countries where a shift in the age distribution towards older age groups has been widely observed.^28^

Widening socioeconomic discrepancy in comorbidity burden, AF incidence and mortality warrant prevention strategies and health-care resource utilisation targeted towards the most deprived individuals.^25^ The increasing population of older patients with AF and comorbidities will mean a greater number of patients at elevated risk of stroke - further increasing the burden on health services. We found that amongst older populations diagnosis was more likely to be made incidentally during a hospital admission, suggesting that the burden of undiagnosed AF in this population may be high. One route to avoid a catastrophic rise in the number of strokes may be through screening for AF in the community. However the evidence base is discordant,^29^ and there is evidence that people who are more comorbid or living in greater deprivation are less likely to participate in screening opportunities.^30^

A strength of this study is the depth, scale and generalisability based on using a well-validated primary care database with linkage to hospital records across all adults and representative of a national population. The results may hence be applicable to a large unselected clinical population. The length of the study period and the number of cases in the study enables both overall and subpopulation analyses. Moreover in view of the similarity in trends of comorbidities and population ageing across European countries, North America and Australasia, our findings are likely to be applicable to many high income countries.^28^

Some limitations exist in our analysis. Our data did not have complete information on type of AF (e.g. paroxysmal, persistent or permanent) in general practice records, precluding interrogation of trends by type of AF; and we could not exclude secondary causes of AF. We could not provide a quantitative analysis of whether increased utilisation of ECG monitoring contributed to increasing AF incidence. The cases reflect identified cases - thus patients with undiagnosed, or whose diagnosis is not recorded, will not be included - and coding practices may change over time. It is possible that some patients were not true incident cases, but we mitigated this through the requirement for cases to have no diagnosis of AF recorded in the 13 years preceding cohort entry and by excluding cases identified within one year of a patient newly registering at a practice. We included atrial flutter diagnosis codes within the outcome measure - in accordance with the majority of other population studies of AF incidence^3^^,^4^,^^6^^,^^9^^,^^11^^,^^12^^,^^14^ as atrial flutter also confers an elevated stroke risk, shares the same recommendations for anticoagulation as AF, and many people with atrial flutter also have AF.^1^

Our findings have implications for health-care resource planning and prevention strategies. The increase in standardised AF incidence across all age groups and socioeconomic strata correlates with increasing burden of comorbidities in the general population, which presents a modifiable target for health-care policy. Initiatives to improve detection of AF and the explosion of acceptable technologies with which to detect AF will also have contributed to increased AF incidence, though the magnitude of this contribution cannot be determined. The increasing comorbidity burden amongst the increasing absolute number of AF patients indicate that management will become more complex and the risk of adverse sequelae will remain high, requiring integrated AF care.^1^ The observed disparities in AF incidence and age of diagnosis by sex and socioeconomic status highlight opportunities for more targeted prevention strategies and resource utilisation to aim for health equity.

## Contributors

JW and CPG conceived and designed the study. JW and CPG contributed to acquiring the data. JW curated the data and conducted the analysis. RN verified the underlying data. RN drafted the Article. All authors provided critical interpretation and revision of the Article. All authors had full access to all the data in the study and accept responsibility to submit for publication.

### Data sharing

Data may be obtained from a third party and are not publicly available. Data used in this study can be accessed through CPRD subject to protocol approval.

## Declaration of interests

CPG reports personal fees from AstraZeneca, Amgen, Bayer, Boehrinher-Ingelheim, Daiichi Sankyo, Vifor, Pharma, Menarini, Wondr Medical, Raisio Group and Oxford University Press. He has received educational and research grants from BMS, Abbott inc., the British Heart Foundation, National Institute of Health Research, Horizon 2020, and from the European Society of Cardiology, outside the submitted work. AJC reports personal fees from Abbott, Bayer, Daiichi Sankyo, Pfizer, BMS, Sanofi, Medtronic, Boston Scientific and Menarini. YMN reports a study grant from Bayer. CW has received a research grant from BMS. All other authors declare no competing interests

## Appendix Supplementary materials

Image, application 1
