# Supplementary material for: Temporal trends and patterns in atrial fibrillation incidence: A population-based study of 3·4 million individuals
Source: Lancet Reg Health Eur. 2022 Apr 25;17:100386. doi: 10.1016/j.lanepe.2022.100386 (PMC9198843; doi:10.1016/j.lanepe.2022.100386)
Supplement: Supplementary file 1 [file mmc1.docx]

**Temporal trends and patterns in atrial fibrillation incidence: a population-based study of 3.4 million individuals**

**Supplementary Material**

[**Supplementary Table S1:** Studies reporting atrial fibrillation incidence in the general population 2](#_Toc98401219)

[**Supplementary Table S2:** Clinical codes used to identify patients with atrial fibrillation. 5](#_Toc98401220)

[**Supplementary Figure S1:** Temporal trends in atrial fibrillation incidence (1998-2017). 6](#_Toc98401221)

[**Supplementary Figure S2:** Temporal trends of proportion of AF diagnosis in hospital setting (1998-2017). 7](#_Toc98401222)

[**Supplementary Figure S3:** Proportion of incident AF diagnosed in hospital setting (a) and proportion of incident AF as primary diagnosis in hospital, by age and sex (b). 8](#_Toc98401223)

[**Supplementary Figure S4:** Temporal trends in age-standardised atrial fibrillation incidence by sex (1998-2017). 9](#_Toc98401224)

[**Supplementary Figure S5**: Temporal trends in atrial fibrillation incidence by socioeconomic status (1998-2017). 10](#_Toc98401225)

[**Supplementary Figure S6**: Temporal trends in age at diagnosis of incident atrial fibrillation by socioeconomic status (1998-2017). 11](#_Toc98401226)

[**Supplementary Figure S7:** Temporal trends in age at diagnosis of incident atrial fibrillation by socioeconomic status and sex (1998-2017). 12](#_Toc98401227)

[**Supplementary Figure S8:** Temporal trends of age and sex-standardised atrial fibrillation incidence in England by regions (1998-2017). 13](#_Toc98401228)

# **Supplementary Table S1:** Studies reporting atrial fibrillation incidence in the general population

| Author | Country | Design /  Data Source | Age range (years) | Exclusions other than preceding AF | Case Definition | Health Sector | Size | Time Period | Reported Crude Incidence  (Rate / 100,000 person-years) | Reported Standardised Incidence  (Rate / 100,000 person-years) | Trend | Standardised | |
| --- | --- | --- | --- | --- | --- | --- | --- | --- | --- | --- | --- | --- | --- |
| Briffa, 2016 (Western Australia)^1^ | Australia | Administrative database | 35 - 84 | ∙∙ | Clinical diagnosis codes | Secondary care | 57 552 | 1995 - 1998 |  | 439 | 🡹 | Census |  |
|  |  |  |  |  |  |  |  | 1999 - 2002 | ∙∙ | 399 |  |  |  |
|  |  |  |  |  |  |  |  | 2003 - 2006 |  | 373 |  |  |  |
|  |  |  |  |  |  |  |  | 2007 - 2010 |  | 383 |  |  |  |
| Chamberlain, 2014  (Olmstead County)^2^ | USA | Administrative database | ≥18 | Post-operative AF | ECG validation or physician diagnosis |  | 3 344 | 2000 |  | 299 | 🡸🡺 |  |  |
|  |  |  |  |  |  | Primary and secondary care |  | 2005 | ∙∙ | 323 |  | Census |  |
|  |  |  |  |  |  |  |  | 2010 |  | 323 |  |  |  |
| Colila, 2013 (Nationwide)^3^ | USA | Health insurance database | ≥20 | Enrolled for less than 1 year, preceding use of warfarin with the exception of a diagnosis of atrial flutter, cardiac surgery during 30 days before AF, hyperthyroidism, rheumatic heart disease, alcohol/substance abuse in the 12 months before AF diagnosis | Clinical diagnosis codes and/or ECG validation when related to a single outpatient visit | Primary and secondary care | 8 749 | 2002 | ∙∙ | 220 | 🡹 | Census |  |
|  |  |  |  |  |  |  |  | 2007 |  | 350 |  |  |  |
| Heeringa, 2006 (Rotterdam)^4^ | Netherlands | Population-based prospective cohort | ≥55 | ∙∙ | ECG, clinical diagnosis codes validated by senior physician | Primary and secondary care | 437 | 1990 - 2000 | 990 | ∙∙ | ∙∙ | ∙∙ |  |
| Hegelund, 2021 (Nationwide)^5^ | Denmark | Registry | ≥18 | ∙∙ | Clinical diagnosis codes | Secondary care | ∙∙ | 2004 |  | 391 | 🡻 | Nordic standard population |  |
|  |  |  |  |  |  |  |  | 2015 | ∙∙ | 481 |  |  |  |
|  |  |  |  |  |  |  |  | 2018 |  | 367 |  |  |  |
| Kjerpeseth, 2020 (Nationwide)^6^ | Norway | Registry | ≥18 | ∙∙ | Clinical diagnosis codes | Secondary care | 175 979 | 2004 | ∙∙ | 433 | 🡸🡺 |  |  |
|  |  |  |  |  |  |  |  | 2004 |  | 440 |  | Census |  |
| Lane, 2017 (Nationwide)^7^ | UK | Electronic health records | ≥18 | Valvular heart disease, past valve intervention | Clinical diagnosis codes | Primary and secondary care | 57 818 | 1998 - 2001 | ∙∙ | 111 | 🡹 |  |  |
|  |  |  |  |  |  |  |  | 2002 - 2006 |  | 133 |  | Census |  |
|  |  |  |  |  |  |  |  | 2007 - 2010 |  | 133 |  |  |  |
| Lehto, 2021 (Nationwide)^8^ | Finland | Registry | ∙∙ | Preceding warfarin prescription in 2004-2007 | Clinical diagnosis codes | Primary and secondary care | 256 323 | 2007 | 330 | - | 🡹 | Nordic standard population |  |
|  |  |  |  |  |  |  |  | 2018 | 456 |  |  |  |  |
| Martinez, 2015 (Nationwide)^9^ | UK | Electronic health records | ≥45 | Less than 1 year of activity in CPRD, no preceding use of anticoagulants, antiarrhythmics, cardioversion, mitral valve disease, valvular heart surgery | Clinical diagnosis codes | Primary and secondary care | 91 707 | 2001 | 560 | 670 | 🡹 | Census |  |
|  |  |  |  |  |  |  |  | 2013 | 640 |  |  |  |  |
| Medonca, 2020 (Nationwide)^10^ | UK | Electronic health records | ≥40 | ∙∙ | Clinical diagnosis codes | Primary care | 99 836 | 2004 - 2018 | ∙∙ | 452 | 🡹 | Denominator |  |
| Miyasaka, 2006 (Olmstead County)^11^ | USA | Administrative database | ≥18 | ∙∙ | ECG validation | Primary and secondary care | 4 618 | 1980 | ∙∙ | 304 | 🡹 | Census |  |
|  |  |  |  |  |  |  |  | 2000 |  | 368 |  |  |  |
| Murphy, 2007 (Nationwide)^12^ | Scotland | Electronic health records |  | ∙∙ | Clinical diagnosis codes | Primary care | - | 2001 - 2002 | ∙∙ | 90 (men)  80 (women) | - | Denominator |  |
| Piccini, 2012 (Nationwide)^13^ | USA | Health insurance database | ≥65 | Research claims | Clinical diagnosis codes | Primary and secondary care | 433 123 | 1993 | ∙∙ | 273 | 🡸🡺 | Census |  |
|  |  |  |  |  |  |  |  | 2007 |  | 283 |  |  |  |
| Renoux, 2014 (Quebec)^14^ | Canada | Administrative database | ∙∙ | Valvular aortic or mitral heart disease, previous valvular repair, hyperthyroidism in the preceding 2 years to cohort entry | Clinical diagnosis codes | Primary and secondary care | 309 556 | 2000 - 2009 | ∙∙ | 324 | 🡹 | Census |  |
| Ruigomez, 2002 (Nationwide)^15^ | UK | Electronic health records | 40 - 89 | Any type but permanent / chronic, enrolled with general practitioner for more than 2 years, and a computerized prescription history of more than 1 year before study start, preceding cancer history or heart rhythm disorder diagnosis | Clinical diagnosis codes, questionnaire responses | Primary care | 1 035 | 1996 | 170 | ∙∙ | ∙∙ | ∙∙ |  |
| Schmidt, 2013 (Nationwide)^16^ | Denmark | Registry | ∙∙ | ∙∙ | Clinical diagnosis codes | Secondary care | 312 420 | 1983 | ∙∙ | 98 | 🡹 | Denominator |  |
|  |  |  |  |  |  |  |  | 2012 |  | 307 |  |  |  |
| Schnabel, 2015 (Framingham)^17^ | USA | Population-based prospective cohort | 50 - 89 | ∙∙ | Cohort follow-up ECG and ECG validation of medical records | Cohort follow-up and medical records | 1 544 | 1958 – 1967 | ∙∙ | 370 (men)  250 (women) | 🡹 | Denominator |  |
|  |  |  |  |  |  |  |  | 1998 – 2007 |  | 1340 (men)  860 (women |  |  |  |
| Scowcroft, 2013 (Nationwide)^18^ | UK | Primary care | ≥18 | Valvular heart disease, past valve intervention, less than 12 months prior records or less than 12 months follow up records | Clinical diagnosis codes | Primary care | 55 847 | 2000 | 127 (men)  121 (women) | - | 🡹 | ∙∙ |  |
|  |  |  |  |  |  |  |  | 2011 | 197 (men)  161 (women) |  |  |  |  |
| Stefansdottir, 2011 (Rekjavik)^19^ | Iceland | Electronic health records | 20 - 99 | Post-operative AF | Clinical diagnosis codes | Secondary care | 4 905 | 1991 – 1993 | ∙∙ | 220 | 🡸🡺 | Denominator |  |
|  |  |  |  |  |  |  |  | 1994 – 1996 |  | 230 |  |  |  |
|  |  |  |  |  |  |  |  | 1997 – 1999 |  | 240 |  |  |  |
|  |  |  |  |  |  |  |  | 2000 – 2002 |  | 230 |  |  |  |
|  |  |  |  |  |  |  |  | 2003 – 2006 |  | 220 |  |  |  |
|  |  |  |  |  |  |  |  | 2006 – 2008 |  | 230 |  |  |  |
| Stewart, 2001 (Renfrew/  Paisley)^20^ | Scotland | Population-based prospective cohorts | 55 - 64 | ∙∙ | ECG validation |  | ∙∙ | 1997 – 2007 | 131 (men) | ∙∙ | ∙∙ | ∙∙ |  |
|  |  |  |  |  |  |  |  |  | 44 (women) |  |  |  |  |
| Vermond, 2015 (PREVEND)^21^ | Netherlands | Population-based prospective cohort | 28 - 75 | Insulin-dependent diabetes mellitus, pregnant women, persons unable or unwilling to participate, no ECG, | Cohort follow- up ECG | Cohort follow-up | 265 | 1997 – 2008 | 330 | ∙∙ | ∙∙ | ∙∙ |  |
| Wilke, 2012 (Nationwide)^22^ | Germany | Health insurance cohort | ≥18 | Not continuously enrolled for 2 years | Clinical diagnosis codes | Primary and secondary care | ∙∙ | 2007 – 2008 | ∙∙ | 436 (men)  387 (women) | ∙∙ | Census |  |
| Williams, 2020 (Pennsylvania)^23^ | USA | Electronic health records | ≥18 | Less than 2 years of usage of health services, at least one EHR encounter | Clinical diagnosis codes | Primary care | 22 077 | 2008 | ∙∙ | 474 | 🡹 | Census |  |
|  |  |  |  |  |  |  |  | 2018 |  | 682 |  |  |  |
| Williams, 2017 (Pennsylvania)^24^ | USA | Electronic health records | ≥18 | Less than 2 years of usage of health services, at least one EHR encounter | Clinical diagnosis codes |  | 21 133 | 2004 | 700 | 470 | 🡹 | Census |  |
|  |  |  |  |  |  | Electronic health records |  | 2008 |  | 500 |  |  |  |
|  |  |  |  |  |  |  |  | 2012 |  | 580 |  |  |  |
|  |  |  |  |  |  |  |  | 2016 |  | 620 |  |  |  |

Abbreviations: AF, atrial fibrillation; ECG, electrocardiogram; GP, general practitioner; UK, United Kingdom; USA, United States of America

Trends: 🡹 indicates increasing trend, 🡸🡺 indicates stable trend, 🡻 indicates declining trend.

# **Supplementary Table S2:** Clinical codes used to identify patients with atrial fibrillation.

| **Coding system** | **Term** | **Code** |
| --- | --- | --- |
| Read code | H/O: atrial fibrillation | 14AN.00 |
| Read code | History of atrial flutter | 14AR.00 |
| Read code | ECG: atrial fibrillation | 3272.00 |
| Read code | ECG: atrial flutter | 3273.00 |
| Read code | Implant intravenous pacemaker for atrial fibrillation | 7936A00 |
| Read code | Referral to atrial fibrillation clinic | 8HTy.00 |
| Read code | Provision of written information about atrial fibrillation | 8OAD.00 |
| Read code | Exception reporting: atrial fibrillation quality indicators | 9hF..00 |
| Read code | Atrial fibrillation and flutter | G573.00 |
| Read code | Atrial fibrillation | G573000 |
| Read code | Atrial flutter | G573100 |
| Read code | Paroxysmal atrial fibrillation | G573200 |
| Read code | Non-rheumatic atrial fibrillation | G573300 |
| Read code | Permanent atrial fibrillation | G573400 |
| Read code | Persistent atrial fibrillation | G573500 |
| Read code | Paroxysmal atrial flutter | G573600 |
| Read code | Atrial fibrillation and flutter NOS | G573z00 |
| ICD-10 | Atrial fibrillation and flutter | I48 |
| ICD-10 | Paroxysmal atrial fibrillation | I48.0 |
| ICD-10 | Persistent atrial fibrillation | I48.1 |
| ICD-10 | Chronic atrial fibrillation | I48.2 |
| ICD-10 | Typical atrial flutter | I48.3 |
| ICD-10 | Atypical atrial flutter | I48.4 |
| ICD-10 | Unspecified atrial fibrillation and atrial flutter | I48.9 |

Read codes were used to identify patients with atrial fibrillation in general practice records and ICD-10 codes to identify patients with atrial fibrillation in hospital discharge records.

# **Supplementary Figure S1:** Temporal trends in atrial fibrillation incidence (1998-2017).


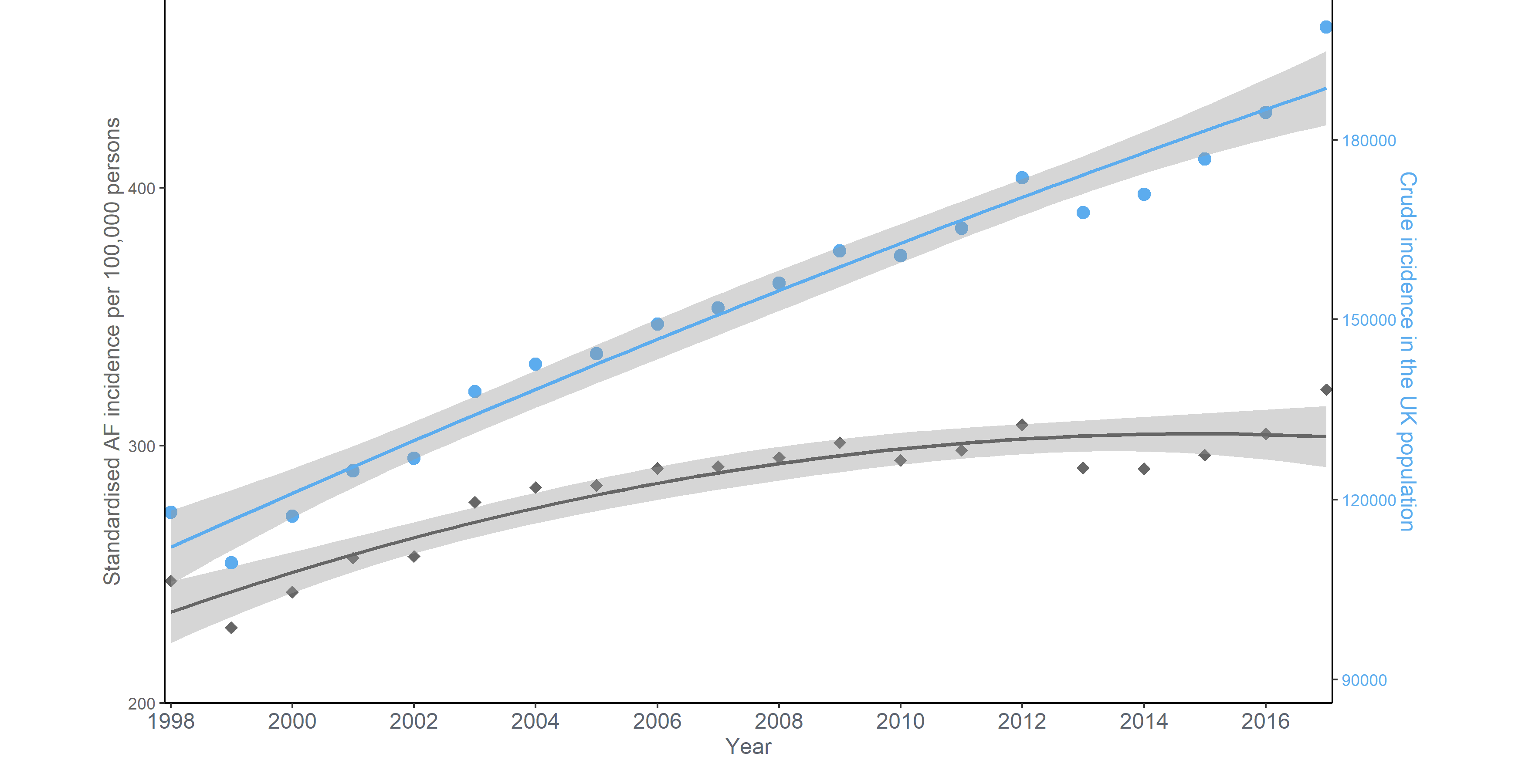


Crude incidence in the UK population is presented as the estimated absolute number of yearly new atrial fibrillation (AF) diagnoses by year (blue line). Standardised atrial fibrillation incidence presents cases per 100,000 persons from the European Standard population (black line) by year.

# **Supplementary Figure S2:** Temporal trends of proportion of AF diagnosis in hospital setting (1998-2017).


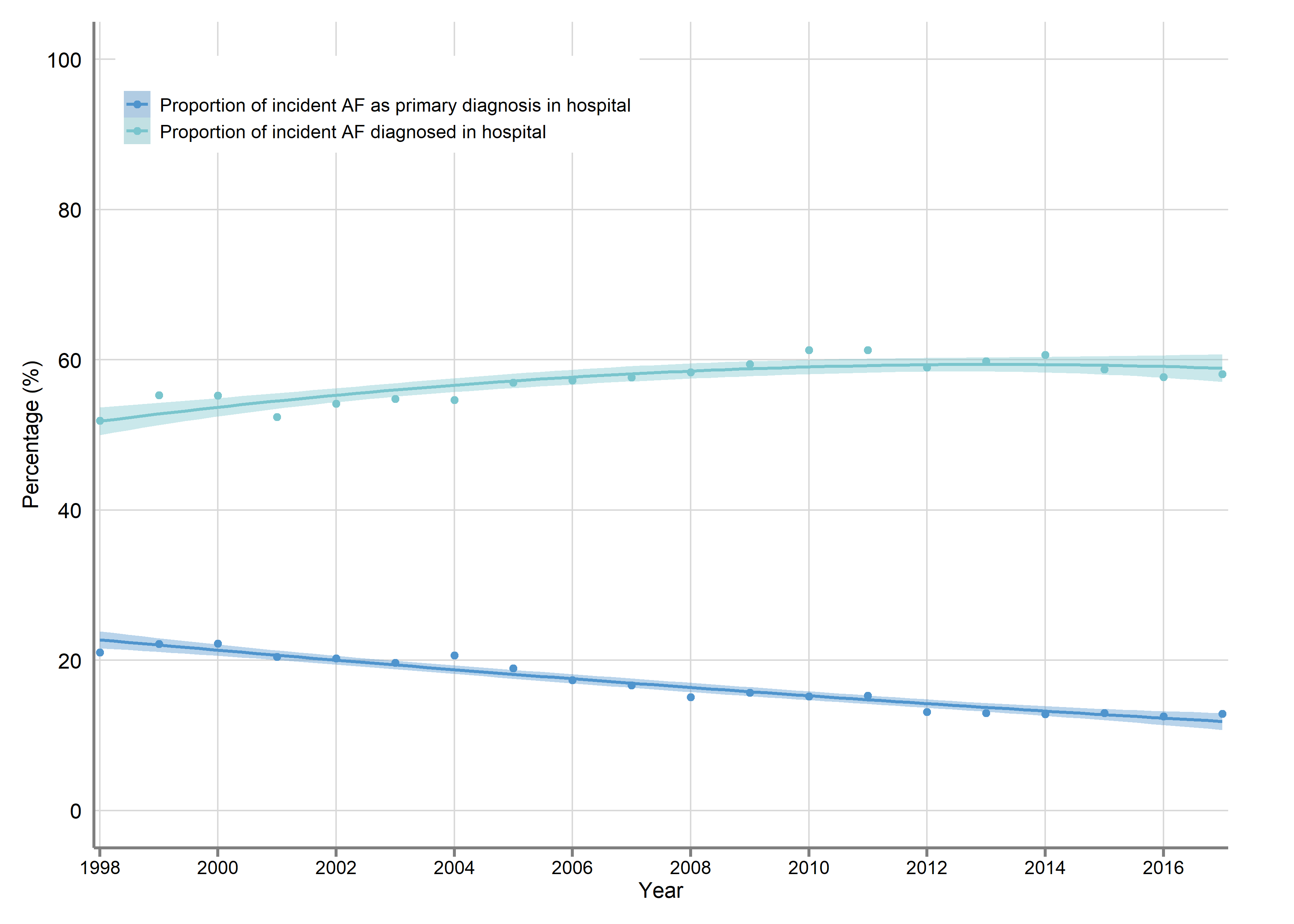


# **Supplementary Figure S3:** Proportion of incident AF diagnosed in hospital setting (A), and proportion of incident AF as primary diagnosis in hospital, by age and sex (B).


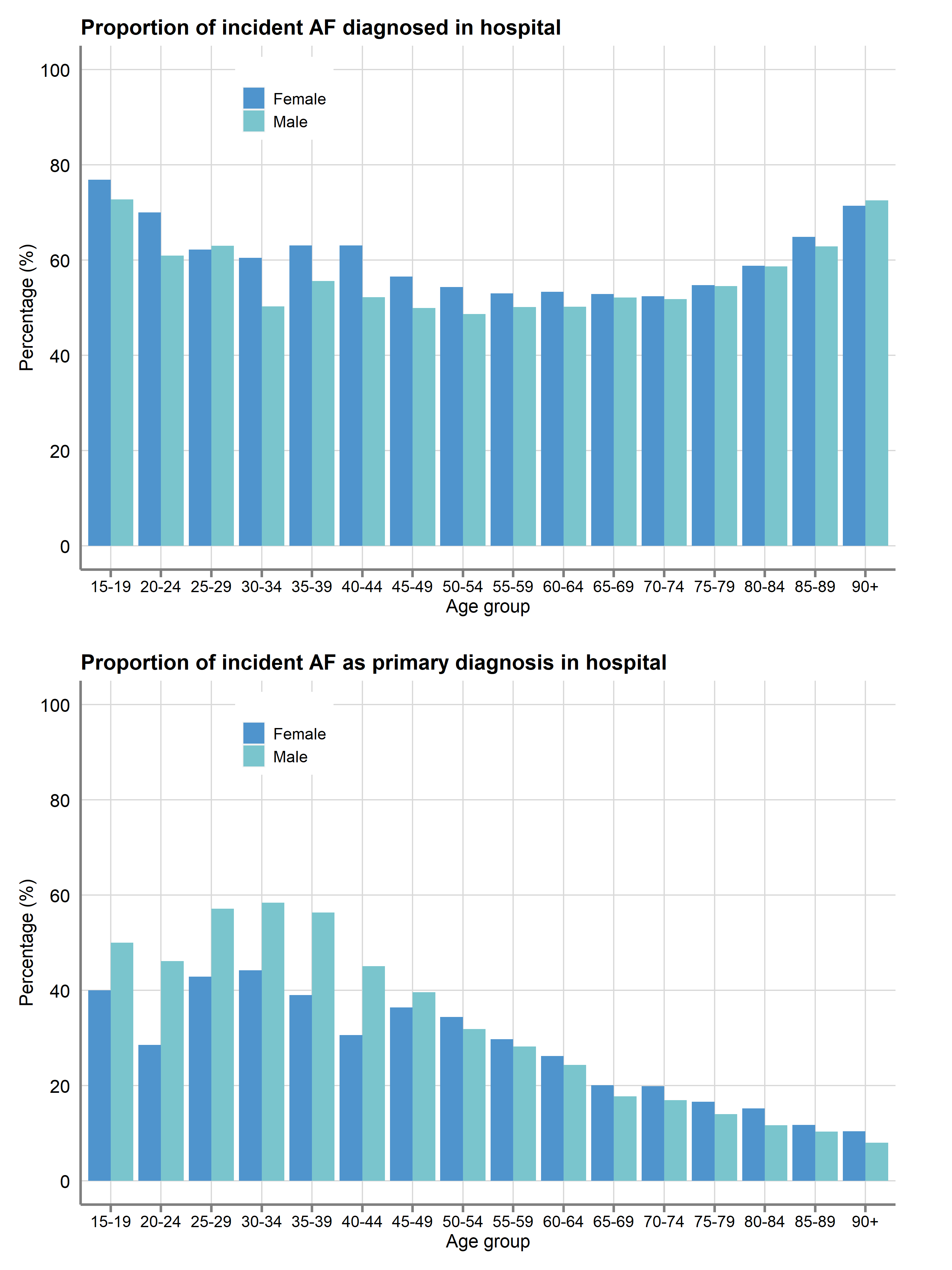


# **Supplementary Figure S4:** Temporal trends in age-standardised atrial fibrillation incidence by sex (1998-2017).


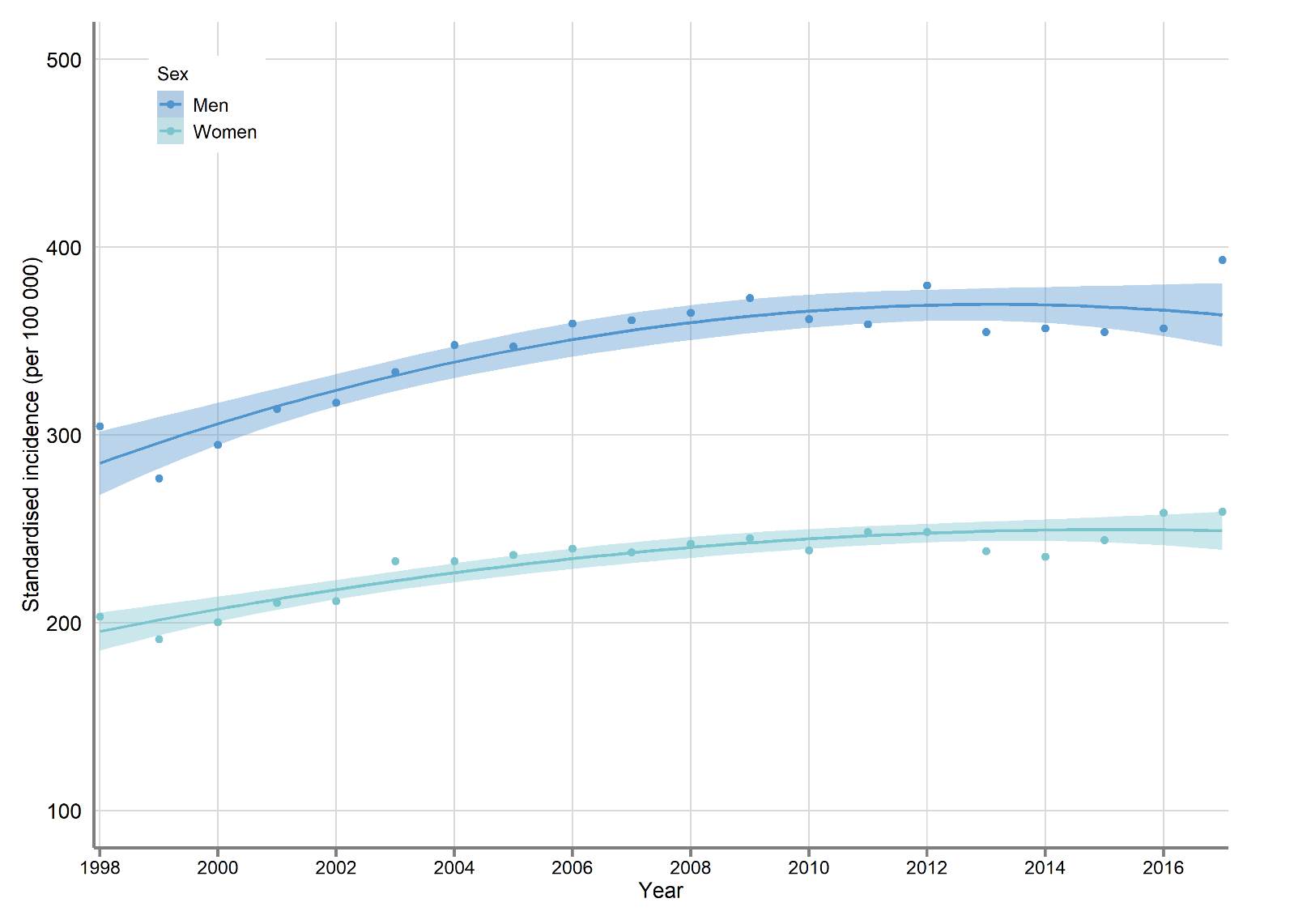


Age-standardised incidence per 100,000 persons by year and sex with fitted local polynomial regression lines and 95% confidence bands.

# **Supplementary Figure S5**: Temporal trends in atrial fibrillation incidence by socioeconomic status (1998-2017).


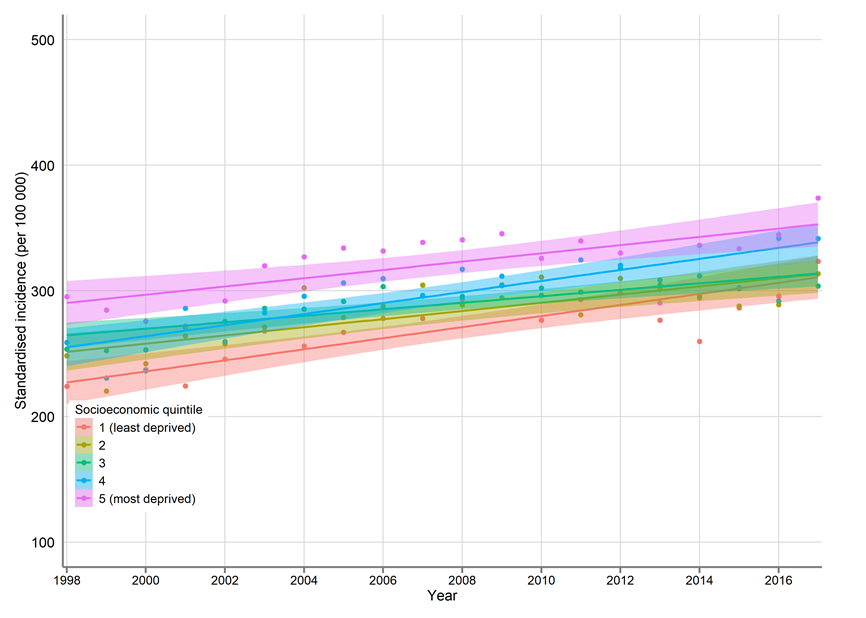


Age and sex-standardised incidence per 100 000 people by year and socioeconomic status are presented with fitted local polynomial regression lines and 95% confidence bands.

# **Supplementary Figure S6**: Temporal trends in age at diagnosis of incident atrial fibrillation by socioeconomic status (1998-2017).


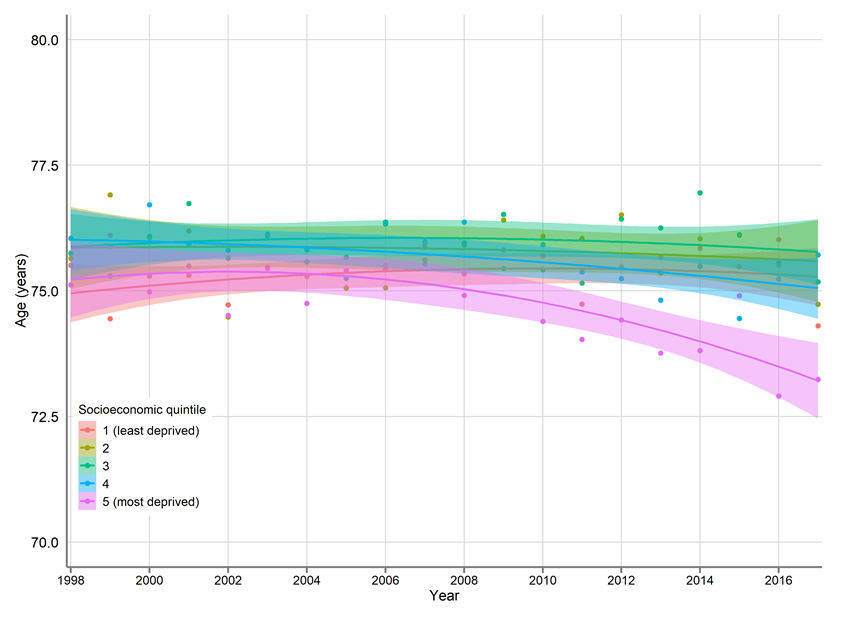


Mean age at incident atrial fibrillation diagnosis by year and socioeconomic quintile is presented with fitted local polynomial regression lines and 95% confidence bands.

# **Supplementary Figure S7:** Temporal trends in age at diagnosis of incident atrial fibrillation by socioeconomic status and sex (1998-2017).


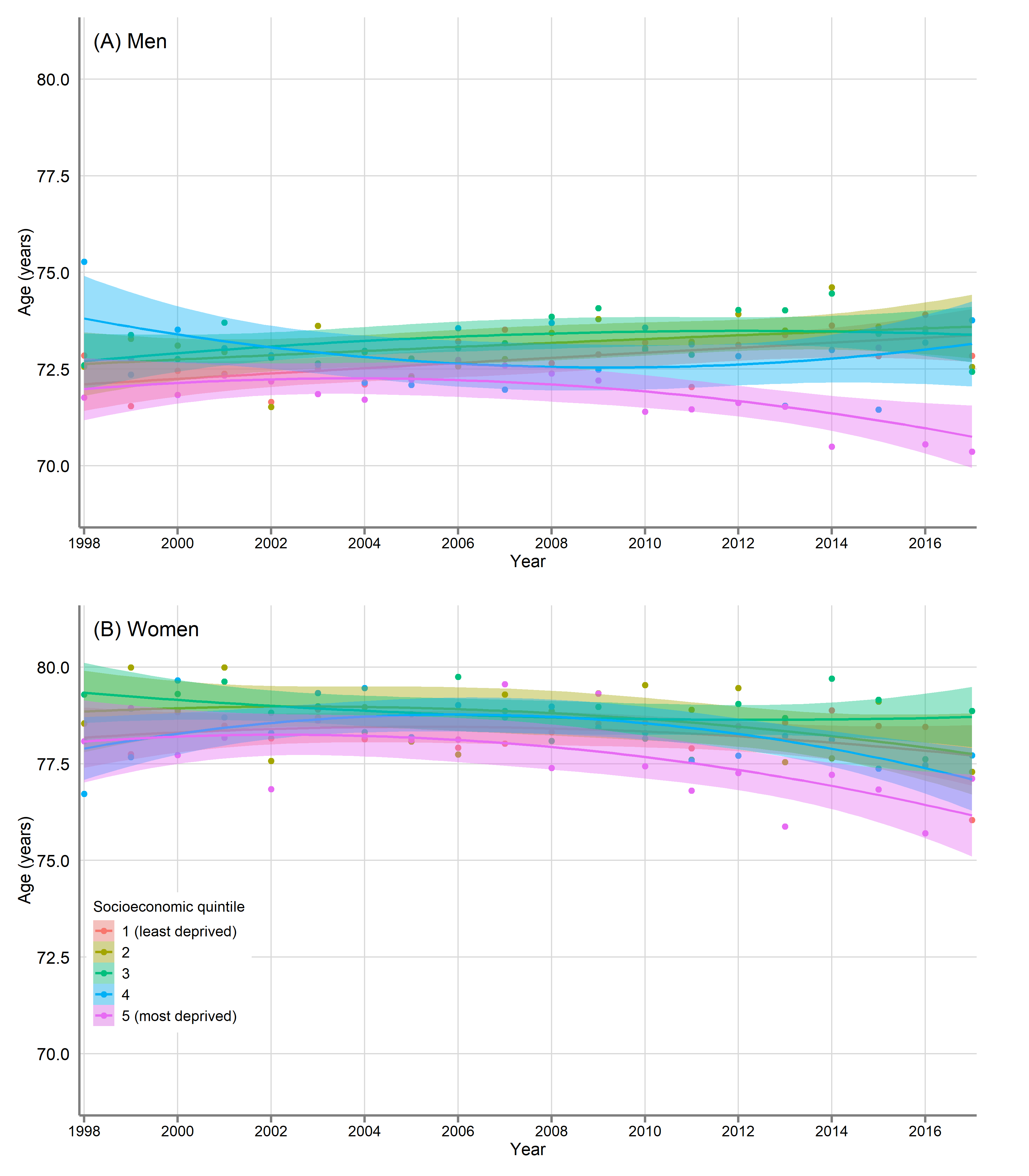


Mean age at incident atrial fibrillation (AF) diagnosis by year and socioeconomic quintile is presented (A) in men and (B) in women with fitted local polynomial regression lines and 95% confidence bands.

# **Supplementary Figure S8:** Temporal trends of age and sex-standardised atrial fibrillation incidence in England by regions (1998-2017).

#


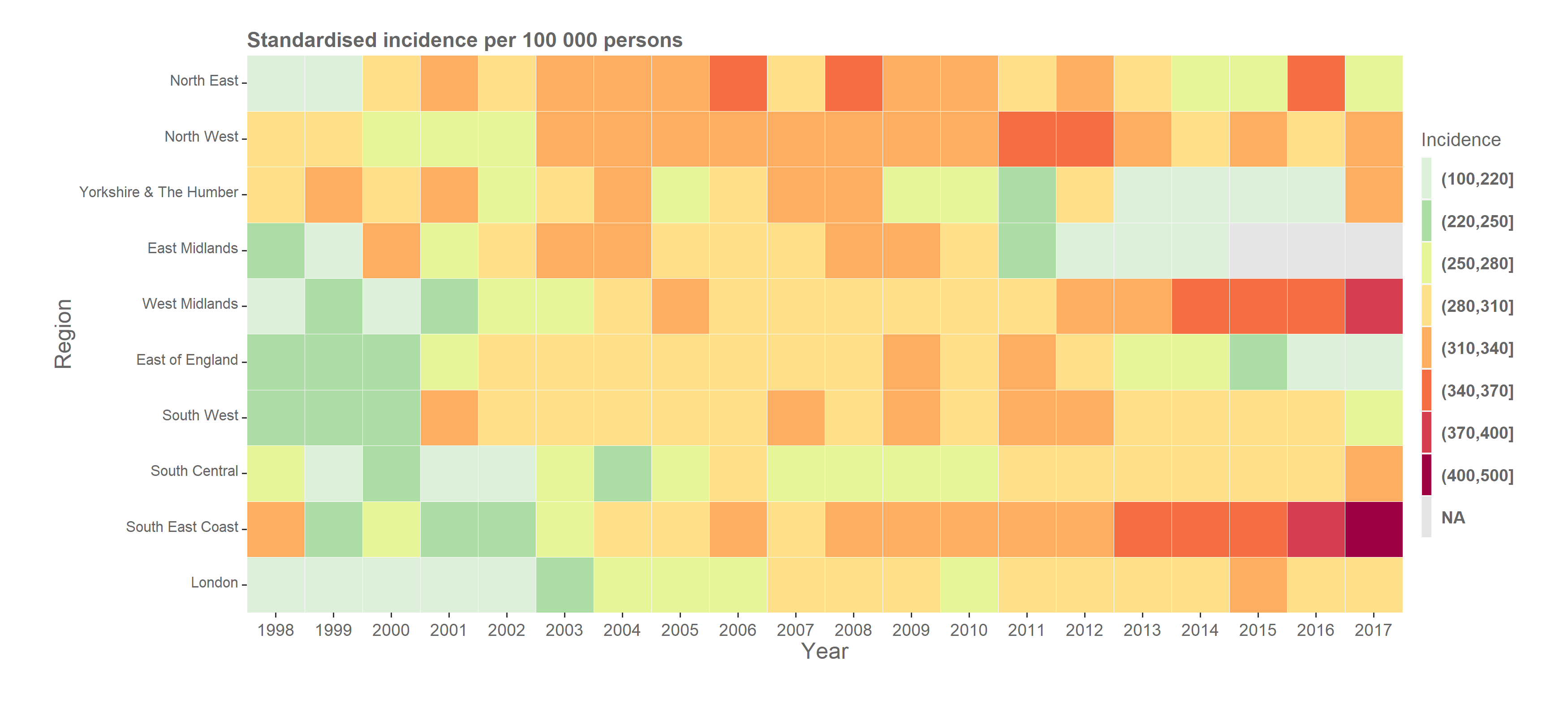


Incidence rates are standardised to the European Standard population. Geographical division refers to the information provided by Clinical Practice Research Datalink (CPRD). Regions are ordered from North to South.

**References**

1. Briffa T, Hung J, Knuiman M, et al. Trends in incidence and prevalence of hospitalization for atrial fibrillation and associated mortality in Western Australia, 1995–2010. *Int J Cardiol* 2016; **208**: 19-25.

2. Chamberlain AM, Gersh BJ, Alonso A, et al. Decade-long trends in atrial fibrillation incidence and survival: a community study. *The American journal of medicine* 2015; **128**(3): 260-7. e1.

3. Colilla S, Crow A, Petkun W, Singer DE, Simon T, Liu X. Estimates of current and future incidence and prevalence of atrial fibrillation in the US adult population. *The American journal of cardiology* 2013; **112**(8): 1142-7.

4. Heeringa J, van der Kuip DA, Hofman A, et al. Prevalence, incidence and lifetime risk of atrial fibrillation: the Rotterdam study. *Eur Heart J* 2006; **27**(8): 949-53.

5. Hegelund E, Kjerpeseth L, Mortensen L, et al. Prevalence and incidence rates of atrial fibrillation in Denmark 2004–2018. *Eur Heart J* 2021; **42**(Supplement_1): ehab724. 0453.

6. Kjerpeseth LJ, Igland J, Selmer R, et al. Prevalence and incidence rates of atrial fibrillation in Norway 2004–2014. *Heart* 2021; **107**(3): 201-7.

7. Lane DA, Skjøth F, Lip GY, Larsen TB, Kotecha D. Temporal trends in incidence, prevalence, and mortality of atrial fibrillation in primary care. *Journal of the American Heart Association* 2017; **6**(5): e005155.

8. Lehto M, Haukka J, Halminen O, et al. Incidence of atrial fibrillation. A comprehensive nationwide analysis in Finland. *Eur Heart J* 2021; **42**(Supplement_1): ehab724. 0464.

9. Martinez C, Katholing A, Wallenhorst C, Granziera S, Cohen AT, Freedman SB. Increasing incidence of non-valvular atrial fibrillation in the UK from 2001 to 2013. *Heart* 2015; **101**(21): 1748-54.

10. Mendonça SC, Saunders CL, Lund J, Mant J, Edwards D. Temporal trends in incidence of atrial fibrillation in primary care records: a population-based cohort study. *BMJ open* 2020; **10**(12): e042518.

11. Miyasaka Y, Barnes ME, Gersh BJ, et al. Secular trends in incidence of atrial fibrillation in Olmsted County, Minnesota, 1980 to 2000, and implications on the projections for future prevalence. *Circulation* 2006; **114**(2): 119-25.

12. Murphy NF, Simpson CR, Jhund PS, et al. A national survey of the prevalence, incidence, primary care burden and treatment of atrial fibrillation in Scotland. *Heart* 2007; **93**(5): 606-12.

13. Piccini JP, Hammill BG, Sinner MF, et al. Incidence and prevalence of atrial fibrillation and associated mortality among Medicare beneficiaries: 1993–2007. *Circ Cardiovasc Qual Outcomes* 2012; **5**(1): 85-93.

14. Renoux C, Patenaude V, Suissa S. Incidence, mortality, and sex differences of non‐valvular atrial fibrillation: a population‐based study. *Journal of the American Heart Association* 2014; **3**(6): e001402.

15. Ruigómez A, Johansson S, Wallander M-A, Rodrı́guez LAGa. Incidence of chronic atrial fibrillation in general practice and its treatment pattern. *J Clin Epidemiol* 2002; **55**(4): 358-63.

16. Schmidt M, Ulrichsen SP, Pedersen L, Bøtker HE, Nielsen JC, Sørensen HT. 30-year nationwide trends in incidence of atrial fibrillation in Denmark and associated 5-year risk of heart failure, stroke, and death. *Int J Cardiol* 2016; **225**: 30-6.

17. Schnabel RB, Yin X, Gona P, et al. 50 year trends in atrial fibrillation prevalence, incidence, risk factors, and mortality in the Framingham Heart Study: a cohort study. *The Lancet* 2015; **386**(9989): 154-62.

18. Scowcroft A, Cowie M. Atrial fibrillation: improvement in identification and stroke preventive therapy—data from the UK Clinical Practice Research Datalink, 2000–2012. *Int J Cardiol* 2014; **171**(2): 169-73.

19. Stefansdottir H, Aspelund T, Gudnason V, Arnar DO. Trends in the incidence and prevalence of atrial fibrillation in Iceland and future projections. *Europace* 2011; **13**(8): 1110-7.

20. Stewart S, Hart C, Hole D, McMurray J. Population prevalence, incidence, and predictors of atrial fibrillation in the Renfrew/Paisley study. *Heart* 2001; **86**(5): 516-21.

21. Vermond RA, Geelhoed B, Verweij N, et al. Incidence of atrial fibrillation and relationship with cardiovascular events, heart failure, and mortality: a community-based study from the Netherlands. *J Am Coll Cardiol* 2015; **66**(9): 1000-7.

22. Wilke T, Groth A, Mueller S, et al. Incidence and prevalence of atrial fibrillation: an analysis based on 8.3 million patients. *Europace* 2013; **15**(4): 486-93.

23. Williams BA, Chamberlain AM, Blankenship JC, Hylek EM, Voyce S. Trends in atrial fibrillation incidence rates within an integrated health care delivery system, 2006 to 2018. *JAMA network open* 2020; **3**(8): e2014874-e.

24. Williams BA, Honushefsky AM, Berger PB. Temporal trends in the incidence, prevalence, and survival of patients with atrial fibrillation from 2004 to 2016. *The American journal of cardiology* 2017; **120**(11): 1961-5.
